# Drug susceptibility of uropathogens isolated from patients treated at the Mazovian Specialized Hospital in Radom

**DOI:** 10.3389/abp.2025.14082

**Published:** 2025-02-27

**Authors:** Zuzanna Trześniewska-Ofiara, Mariola Mendrycka, Agnieszka Woźniak-Kosek

**Affiliations:** ^1^ Department of Laboratory Diagnostics, Mazovian Specialist Hospital Ltd., Radom, Poland; ^2^ Department of Cosmetology, Faculty of Medical Sciences and Health Sciences, Casimir Pulaski University of Radom, Radom, Poland; ^3^ Department of Laboratory Diagnostics, Military Institute of Medicine-National Research Institute, Warsaw, Poland

**Keywords:** uropathogens, urinary tract infections, UTI, drug susceptibility, antibiotic therapy

## Abstract

Urinary tract infections (UTI) are a significant problem among populations worldwide. It is mainly associated with the increasing incidence of recurrence, complications and the increasing drug resistance of uropathogens. The aim of this study was to demonstrate the prevalence of resistance among pathogens causing urinary tract infections. The material for the study was data obtained from the Mazovian Specialized Hospital (M.S.H) in Radom over a period of 2 years. Urine was collected from hospitalized patients with UTI. Statistical calculations were performed using statistical software. During the study period, 3,917 patients underwent microbiological examination of urine, and almost 15% of them were found to be infected with UTI. Based on statistical analysis of drug susceptibility of the most common uropathogens, it was shown that urinary tract infections caused by *Escherichia coli* or *Klebsiella pneumoniae*, among others, often show high resistance to fluoroquinolones and β-lactam antibiotics. *Proteus mirabilis* strains have been shown to be more resistant to aminoglycosides and fluoroquinolones than to beta-lactams. In the case of *Pseudomonas* aeruginisa, resistance to fluoroquinolones predominates. On the other hand, UTI caused by Acientobacter baumannii should be treated based on the results of drug susceptibility testing due to the increasing prevalence of multidrug-resistant strains.

## Introduction

Antibiotics are considered one of the greatest medical discoveries (Moser et al., 2019). Their history dates back to 1928, when natural penicillin was discovered (Grayson, 2010). The widespread use of antibacterial drugs is due to their broad spectrum of action. Despite advances in medicine, antibiotics remain the first-line treatment for infectious diseases (Baran et al., 2023). Proper use of antibiotics saves millions of lives each year, preventing complications. However, infections still cause numerous deaths, even in developed countries with unrestricted access to antibiotics (Moser et al., 2019). Since the discovery of penicillin, many antibacterial drugs have been introduced, but the effectiveness of treating bacterial infections has been steadily decreasing. This phenomenon is caused by factors such as improper antibiotic selection for patients, incorrect dosing that hinders the achievement of therapeutic concentrations at the site of infection, and the failure to update knowledge about antibiotics, their mechanisms of action, and pharmacotherapy (Grayson, 2010; Bradley, 2018). Modern antibiotic therapy presents a significant challenge for both clinicians and microbiologists due to the growing antimicrobial resistance of pathogens. As a consequence, there are frequent therapeutic failures and rising treatment costs (Woroń, 2020; Sapilak, 2020). Microbial resistance to antibiotics poses a serious threat to the control of infectious diseases. Despite the growing demand for new therapies, the development of new antimicrobial drugs has significantly slowed, mainly due to high costs and the limited profitability of the market (Hatfull, Dedrick, and Schooley, 2022). Among infections that present a significant clinical challenge, urinary tract infections (UTIs) draw particular attention. First documented in ancient Egypt in 1550 BC, they continue to be among the most common bacterial infections worldwide. UTIs affect nearly 150 million people annually worldwide, with an annual incidence of 12% in women and 3% in men (Kuo and Jhang, 2017). UTIs affect patients of all ages and are one of the leading causes of morbidity and mortality among the old adults, accounting for 15.5% of hospitalizations and 6.2% of deaths among people aged 65 and older (Zalewska-Piątek and Piątek, 2020; Flores-Mireles et al., 2015; Cassini et al., 2016; Dubbs and Sommerkamp, 2019). UTIs can be caused by various bacteria and fungi. The most common pathogen is uropathogenic *Escherichia coli* (UPEC), responsible for both complicated and uncomplicated urinary tract infections (Zalewska-Piątek and Piątek, 2020; Trześniewska-Ofiara et al., 2022; Neugent et al., 2020). Other frequently encountered microorganisms include *Klebsiella pneumoniae*, *Enterococcus faecalis*, and *Proteus mirabilis*. Public perception of UTI treatment is that it is an easily treatable condition with antibiotic therapy (Foxman, 2014; Waller et al., 2018). However, problems remain with the chronic nature of these infections, their recurrent character, numerous complications, and rising treatment costs due to increasing resistance in uropathogens (Zalewska-Piątek and Piątek, 2020; Trześniewska-Ofiara et al., 2022; Gaitonde, Malik, and Zimmern, 2019). In recent years, the number of antibiotic-resistant pathogens has increased significantly, making the development of new methods for treating UTIs one of the priorities of modern medicine (Chegini et al., 2021). Urinary tract infections can lead to pyelonephritis and life-threatening urosepsis. Some cases of recurrent UTIs can persist for years and, due to antimicrobial resistance, may require bladder removal (Flores-Mireles et al., 2015; Neugent et al., 2020). This issue requires further research and the development of new therapeutic strategies to effectively address the growing challenges of treating urinary tract infections.

The aim of this study was to analyze the frequency of antibiotic resistance among uropathogens in urinary tract infections in hospitalized patients at the Mazowieckie Specialist Hospital (MSH) in Radom (Poland) in the years 2020–2021.

## Materials and methods

This study utilized data obtained from the Mazowieckie Specialist Hospital (M.S.H.) in Radom, Poland. The analysis covers the period from 1 January 2020, to 31 December 2021. The data consist of results from urine culture tests performed at the Laboratory Diagnostics Department of M.S.H. for samples submitted by patients hospitalized during the study period. The diagnostic criterion for urinary tract infection (UTI) was the presence of clinical symptoms, with the final diagnosis made by the attending physician. Common symptoms observed in patients included dysuria, nocturia, and pyuria.

At the Laboratory Diagnostics Department, urine samples collected for microbiological testing were cultured using the Hoeprich method on two types of media: blood agar with 5% sheep blood and MacConkey agar. Disposable plastic inoculating loops with capacities of 0.01 mL and 0.001 mL (for 10^2^ and 10^3^ dilutions, respectively) were used for inoculation. The cultures were incubated at 35°C–37°C for 24–48 h. The results were reported as the number of microbial cells grown per 1 mL of urine. The microbiological criterion for diagnosing a UTI was bacteriuria of ≥10^4^ CFU/mL.

The next step involved the identification and antimicrobial susceptibility testing of pathogenic microorganisms using the Phoenix M50 system by Becton Dickinson. In some cases, additional E-Test strips on Mueller-Hinton agar were used to verify antimicrobial resistance in uropathogens or to explore alternative therapeutic options.

During the analyzed period, the hospital had 613 beds spread across 21 departments and sub-departments. Data were collected from 20 of these departments, including: Clinical Department of Internal Medicine I, Clinical Department of General Surgery, Clinical Department of Oncological Surgery, Clinical Department of Oncology, Clinical Department of Neurology, Clinical Department of Otolaryngology, Clinical Department of Rehabilitation, Clinical Department of Pediatrics, Obstetrics and Gynecology Department, Trauma and Orthopedic Surgery Department, Cardiology Department, Hematology Department, Pulmonology and Pulmonary Oncology Department, Intensive Care Unit, Rheumatology Department, Internal Medicine II Department, Clinical Neurosurgery Department, Pediatric Surgery Department, Cardiac Surgery Department, Neonatology Department, and Ophthalmology Department. Data from the Emergency Department were excluded from the statistical analysis due to the short length of patient stays in that department.

The study was conducted with the approval of the Bioethics Committee of the University of Technology and Humanities (KB/17/2022) on 10 May 2022.

### Statistical analysis

In order to verify the difference between antibiotic groups in the frequency of drug resistance of pathogens such as *Klebsiella pneumonia* and *Escherichia coli*, the χ^2^ test was used. Additionally, pairwise comparisons were performed using the χ^2^ test to examine the differences between the analysed antibiotics group more precisely. When expected cell frequencies were five or below in 2 × 2 contingency tables, Yates’ correction was carried out. Adjusted standardised residuals were employed to scrutinise differences more precisely, particularly when one variable had more than two categories (Agresti, 2007). In this context, absolute values of adjusted standardised residuals exceeding 1.96 indicated statistically significant results. It should be noted that the difference analyses incorporated data on the number of urine tests conducted. Considering the sample size, the Fisher-Freeman-Halton test (Freeman and Halton, 1951) was used to examine the difference between antibiotic groups in the frequency of drug resistance of the pathogens such as *Enterococcus faecalis, Enterococcus faecium, Proteus mirabilis, Pseudomonas aeruginosa* and *Acinetobacter baumannii*. The pairwise comparisons were performed using the Fisher-Freeman-Halton test (Freeman and Halton, 1951) to examine the differences between the analysed antibiotic groups more precisely. Considering that there was no drug resistance category such as susceptible - increased exposure (I) for *Enterococcus faecalis* and *Enterococcus faecium*, the statistical analysis included drug resistance categories such as susceptible - standard dosing regimen (S) and resistant (R). Additionally, pairwise comparisons were performed using the Fisher exact test (Fisher, 1922) in case of *Enterococcus faecalis*and *Enterococcus faecium*. The antibiotics were grouped into the following groups: 1) β-Lactams (Amoxicillin/Clavulanate, Ampicillin, Cefepime, Cefotaxime, Ceftazidime, Ceftriaxone, Cefuroxime, Ertapenem, Imipenem, Meropenem, and Piperacillin/Tazobactam), 2) Aminoglycosides (Amikacin, Gentamicin, and Tobramycin), 3) Fluoroquinolones (Ciprofloxacin, Levofloxacin, and Norfloxacin), 4) Fosfomycin G6PD, 5) Glycopeptides (Teicoplanin and Vancomycin) and 6) Fosfomycin. It should be noted that different pathogens had different numbers of antibiotic groups. Considering pairwise comparisons, the Bonferroni-adjusted p-value was used to minimise the type I error in the pairwise comparisons. More specifically, the Bonferroni-adjusted p value was 0.05/8 = 0.00625 for *Escherichia coli*, 0.05/4 = 0.0125 for *Klebsiella pneumonia*, 0.05/3 = 0.0167 for *Enterococcus faecalis, Enterococcus faecium, Proteus mirabilis, Pseudomonas aeruginosa* and *Acinetobacter baumannii.* It should be noted that drug resistance data from 2020 to 2021 were also merged for this analysis. Difference analyses included data on the number of urine tests performed.

The effect size for all analyses was computed using φ (Fritz et al., 2012) or Cramér’s V (Cramér, 1961). In this context, the interpretation of effect sizes (φ and Cramér’s V) followed the guidelines proposed by Rea and Parker (1992): 1) from 0 to 0.1 (negligible), 2) from 0.1 to 0.2 (weak), 3) from 0.2 to 0.4 (moderate), 4) from 0.4 to 0.6 (relatively strong), 5) from 0.6 to 0.8 (strong), and 6) from 0.8 to 1.0 (very strong). The statistical software IBM SPSS version 28 was used for statistical analyses.

## Results

There were 31,635 hospitalisations in 2020 and 32,721 in 2021 at the analysed hospital wards. Urinalysis was performed on 1,885 patients in 2020 and 2,032 patients in 2021. Among patients with a urinalysis performed in 2020, 351 (18.62%) had a urinary tract infection. Among patients who had a urinalysis performed in 2021, 236 (11.61%) had a urinary tract infection. There were 2,919 urine tests carried out in 2020 and 2,951 in 2021. Among urine tests performed in 2020, 1,071 (36.69%) urine tests were positive. Among urine tests performed in 2021, 999 (33.85%) urine tests were positive. Moreover, among positive urine tests carried out in 2020, 484 (45.19%) positive urine tests were obtained 72 h after admission to the hospital ward. Additionally, among positive urine tests carried out in 2021, 444 (44.44%) positive urine tests were obtained 72 h after admission to the hospital ward.

### Escherichia coli

In the case of drug resistance of *Escherichia coli* to groups of antibiotics (see Table 1), the findings showed statistically significant differences between these groups of antibiotics in drug resistance frequency of *Escherichia coli* [χ^2^ (df = 8) = 833.82, p < 0.001, Cramér’s V = 0.197]. The effect size was weak (see Table 1). Comparisons between each pair of antibiotic groups were conducted to verify the differences between the analysed antibiotic groups more precisely. In this context, there was a statistically significant difference between Aminoglycosides and β-Lactams antibiotics group in drug resistance frequency of *Escherichia coli* [χ^2^ (df = 2) = 295.48, p < 0.001, Cramér’s V = 0.187]. Considering the adjusted standardised residual values (z > 8.9 and p < 0.001 for all), it can be pointed out that the β-Lactams antibiotics group was more often drug-resistant to *Escherichia coli* than the Aminoglycosides antibiotics group. Additionally, the frequency of *Escherichia coli* susceptibility (increased exposure) was higher for β-Lactams than for the Aminoglycosides antibiotics group. It should be noted that the effect size was weak (see Table 1). The results also presented a statistically significant difference between the β-Lactams and Fluoroquinolones antibiotics group in drug resistance frequency of *Escherichia coli* [χ^2^ (df = 2) = 246.49, p < 0.001, Cramér’s V = 0.172]. Considering the adjusted standardised residual values (z > 5.7 and p < 0.001 for all), it can be pointed out that the Fluoroquinolones antibiotics group was more often drug-resistant of *Escherichia coli* than the β-Lactams antibiotics group. In contrast, the frequency of *Escherichia coli* susceptibility (increased exposure) was higher for β-Lactams than for the Fluoroquinolones antibiotics group. It should be emphasised that the effect size was weak (see Table 1). There was also a statistically significant difference between β-Lactams and Fosfomycin G6PD antibiotics in drug resistance frequency of *Escherichia coli* [χ^2^ (df = 2) = 200.30, p < 0.001, Cramér’s V = 0.169]. Taking into account the adjusted standardised residual values (z > 9.3 and p < 0.001 for all), it can be observed that the β-Lactams antibiotics group was more often drug-resistant of *Escherichia coli* than Fosfomycin G6PD antibiotics. However, the effect size was weak (see Table 1). Similarly, the findings showed a statistically significant difference between the β-Lactams and Fosfomycin antibiotics in drug resistance frequency *Escherichia coli* [χ^2^ (df = 2) = 17.06, p < 0.001, Cramér’s V = 0.050]. Considering the adjusted standardised residual values (z > 2.5 and p < 0.012 for all), it can be pointed out that the β-Lactams antibiotics group was more often drug-resistant of *Escherichia coli* than the Fosfomycin antibiotics. However, it should be highly emphasised that the effect size was negligible (see Table 1). The pairwise comparisons analysis also showed the difference between Aminoglycosides and Fluoroquinolones antibiotics group in drug resistance frequency of *Escherichia coli* [χ^2^ (df = 2) = 346.51, p < 0.001, Cramér’s V = 0.330]. The effect size was moderate. Considering the adjusted standardised residual values (z > 6.2 and p < 0.001 for all), it can be indicated that the Fluoroquinolones antibiotics group was more often drug-resistant of *Escherichia coli* than the Aminoglycosides antibiotics group (see Table 1). For drug resistance frequency of *Escherichia coli*, there was a statistically significant difference between Aminoglycosides and Fosfomycin G6PD antibiotics [χ^2^ (df = 2) = 21.67, p < 0.001, Cramér’s V = 0.095]. Considering the adjusted standardised residual values, differences between these antibiotics groups were presented for drug resistance categories such as susceptible - standard dosing regimen (S; z = 4.6; p < 0.001) and resistant (R; z = 4.3; p < 0.001). More precisely, the Aminoglycosides antibiotics group was more often drug-resistant of *Escherichia coli* than Fosfomycin G6PD. However, it should be highly highlighted that the effect size was negligible (see Table 1). There was a statistically significant difference between Fluoroquinolones and Fosfomycin G6PD antibiotics in drug resistance frequency of *Escherichia coli* [χ^2^ (df = 2) = 259.12, p < 0.001, Cramér’s V = 0.340]. The effect size was moderate. Taking into account the adjusted standardised residual values (z > 4.9 and p < 0.001 for all), it can be noted that the Fluoroquinolones antibiotics group was more often drug-resistant of *Escherichia coli* than Fosfomycin G6PD antibiotics (see Table 1). The pairwise comparisons analysis also showed that the difference between the Fluoroquinolones and Fosfomycin antibiotics group in drug resistance frequency of *Escherichia coli* was statistically significant [χ^2^ (df = 2) = 23.93, p < 0.001, Cramér’s V = 0.124]. Considering the adjusted standardised residual values, differences between these antibiotics groups were presented for drug resistance categories such as susceptible - standard dosing regimen (S; z = 4.9; p < 0.001) and resistant (R; z = 4.5; p < 0.001). More precisely, the Fluoroquinolones antibiotics group was more often drug-resistant of*Escherichia coli* than the Fosfomycin antibiotics. However, it should be highlighted that the effect size was weak (see Table 1). The detailed results are shown in Table 1.

**TABLE 1:** Difference between antibiotics group in drug resistance frequency for *Escherichia coli*.

Drug resistance	Aminoglycosides		β-Lactams		Fluoroquinolones		Fosfomycin w/G6PD		Fosfomycin		χ^2^	p	V
	N	Percent	N	Percent	N	Percent	N	Percent	N	Percent			
S	1,580	93.94%	5,132	75.60%	1,027	68.56%	731	98.25%	53	100.00%	833.82	0.001	0.197
I	6	0.36%	735	10.83%	48	3.20%	0	0.00%	0	0.00%			
R	96	5.71%	921	13.57%	423	28.24%	13	1.75%	0	0.00%			

Pairwise comparisons															
Drug resistance	Aminoglycosides		β-Lactams		χ^2^	p	V	Drug resistance	β-Lactams		Fluoroquinolones		χ^2^	p	V
	N	Percent	N	Percent					N	Percent	N	Percent			
S	1,580	93.94%	5,132	75.60%	295.48	0.001	0.187	S	5,132	75.60%	1,027	68.56%	246.49	0.001	0.172
I	6	0.36%	735	10.83%				I	735	10.83%	48	3.20%			
R	96	5.71%	921	13.57%				R	921	13.57%	423	28.24%			

Drug resistance	β-Lactams		Fosfomycin w/G6PD		χ^2^	p	V	Drug resistance	β-Lactams		Fosfomycin		χ^2^	p	V
	N	Percent	N	Percent					N	Percent	N	Percent			
S	5,132	75.60%	731	98.25%	200.30	0.001	0.163	S	5,132	75.60%	53	100.00%	17.06	0.001	0.050
I	735	10.83%	0	0.00%				I	735	10.83%	0	0.00%			
R	921	13.57%	13	1.75%				R	921	13.57%	0	0.00%			

Drug resistance	Aminoglycosides		Fluoroquinolones		χ^2^	p	V	Drug resistance	Aminoglycosides		Fosfomycin w/G6PD		χ^2^	p	V
	N	Percent	N	Percent					N	Percent	N	Percent			
S	1,580	93.94%	1,027	68.56%	346.51	0.001	0.330	S	1,580	93.94%	731	98.25%	21.67	0.001	0.095
I	6	0.36%	48	3.20%				I	6	0.36%	0	0.00%			
R	96	5.71%	423	28.24%				R	96	5.71%	13	1.75%			

Drug resistance	Aminoglycosides		Fosfomycin		χ^2^	P	V	Drug resistance	Fluoroquinolones		Fosfomycin w/G6PD		χ^2^	p	V
	N	Percent	N	Percent					N	Percent	N	Percent			
S	1,580	93.94%	53	100.00%	3.42	0.181	0.044	S	1,027	68.56%	731	98.25%	259.12	0.001	0.340
I	6	0.36%	0	0.00%				I	48	3.20%	0	0.00%			
R	96	5.71%	0	0.00%				R	423	28.24%	13	1.75%			

Drug resistance	Fluoroquinolones		Fosfomycin		χ^2^	P	V	Drug resistance^a^	Fosfomycin w/G6PD		Fosfomycin		χ^2^	p	φ
	N	Percent	N	Percent					N	Percent	N	Percent			
S	1,027	68.56%	53	100.00%	23.93	0.001	0.124	S	731	98.25%	53	100.00%	0.17	0.682	0.034
I	48	3.20%	0	0.00%				I	0	0.00%	0	0.00%			
R	423	28.24%	0	0.00%				R	13	1.75%	0	0.00%			

Note: S, Susceptible, standard dosing regimen; I, Susceptible, increased exposure; R, Resistant; V, Cramér’s V.

^a^ Due to the lack of drug resistance category I, statistical analyses were performed considering only drug resistance categories S and R.

### Enterococcus faecalis

In the case of drug resistance of *Enterococcus faecalis* to the antibiotics group (see Table 2), the findings showed statistically significant differences between these groups of antibiotics in drug resistance frequency of *Enterococcus faecalis* [Fisher-Freeman-Halton test = 444.01, p < 0.001, Cramér’s V = 0.533]. The effect size was relatively strong (see Table 2). To verify the differences between the analysed antibiotics group more precisely, comparisons between each pair of antibiotics groups were carried out using the Fisher exact test (Fisher, 1922). The pairwise comparisons analysis showed the difference between Aminoglycosides and Glycopeptides antibiotics group in drug resistance frequency of *Enterococcus faecalis* [Fisher’s exact test p < 0.001, φ = 0.541]. The effect size was relatively strong. Based on the results obtained, it can be concluded that the Aminoglycosides antibiotics group was more often drug-resistant of *Enterococcus faecalis* than the Glycopeptides antibiotics group (see Table 2). Similarly, it was demonstrated that the Fluoroquinolones antibiotics group was more often drug-resistant of *Enterococcus faecalis* than the Glycopeptides antibiotics group [Fisher’s exact test p < 0.001, φ = 0.599]. The detailed results are shown in Table 2.

**TABLE 2:** Difference between antibiotics group in drug resistance frequency for *Enterococcus faecalis*.

Drug resistance	Aminoglycosides		Fluoroquinolones		Glycopeptides		Fisher-Freeman-Halton exact test	p	V
	N	Percent	N	Percent	N	Percent			
S	246	50.62%	92	45.54%	649	96.58%	444.01	0.001	0.533
R	240	49.38%	110	54.46%	23	3.42%			

Pairwise comparisons						
Drug resistance	Aminoglycosides		Fluoroquinolones		Fisher’s exact test p value	φ
	N	Percent	N	Percent		
S	246	50.62%	92	45.54%	0.242	0.046
R	240	49.38%	110	54.46%		

Drug resistance	Aminoglycosides		Glycopeptides		Fisher’s exact test p value	φ
	N	Percent	N	Percent		
S	246	50.62%	649	96.58%	0.001	0.541
R	240	49.38%	23	3.42%		

Drug resistance	Fluoroquinolones		Glycopeptides		Fisher’s exact test p value	φ
	N	Percent	N	Percent		
S	92	45.54%	649	96.58%	0.001	0.599
R	110	54.46%	23	3.42%		

Note: S, Susceptible, standard dosing regimen; I, Susceptible, increased exposure; R, Resistant; V, Cramér’s V.

### Enterococcus faecium

For drug resistance of *Enterococcus faecium* to antibiotics group, the findings showed statistically significant differences between these groups of antibiotics in drug resistance frequency of *Enterococcus faecium* [Fisher-Freeman-Halton test = 230.33, p < 0.001, Cramér’s V = 0.591]. The effect size was relatively strong (see Table 3). To verify the differences between the analysed antibiotics group more precisely, comparisons between each pair of antibiotics groups were carried out using the Fisher exact test (Fisher, 1922). The pairwise comparisons analysis showed the difference between the Aminoglycosides and Fluoroquinolones antibiotics group in the drug resistance frequency of *Enterococcus faecium* [Fisher’s exact test p < 0.001, φ = 0.497]. The effect size was relatively strong. Based on the results obtained, it can be concluded that the Fluoroquinolones antibiotics group was more often drug-resistant of *Enterococcus faecium* than the Aminoglycosides antibiotics group (see Table 3). Similarly, it was observed thattheFluoroquinolones antibiotics group was more often drug-resistant of *Enterococcus faecium* than the Glycopeptides antibiotics group [Fisher’s exact test p < 0.001, φ = 0.697]. Additionally, there was a statistically significant difference between the Aminoglycosides and Glycopeptides antibiotics group in the drug resistance frequency of *Enterococcus faecalis* [Fisher’s exact test p < 0.001, φ = 0.497]. In this context, the Aminoglycosides antibiotics group was more often drug-resistant of *Enterococcus faecium* than the Glycopeptides antibiotics group (see Table 3). The detailed results are shown in Table 3.

**TABLE 3:** Difference between antibiotics group in drug resistance frequency for *Enterococcus faecium*.

Drug resistance	Aminoglycosides		Fluoroquinolones		Glycopeptides		Fisher-Freeman-Halton exact test	p	V
	N	Percent	N	Percent	N	Percent			
S	86	50.89%	0	0.00%	285	83.58%	230.33	0.001	0.591
R	83	49.11%	78	100.00%	56	16.42%			

Pairwise comparisons						
Drug resistance	Aminoglycosides		Fluoroquinolones		Fisher’s exact test p value	φ
	N	Percent	N	Percent		
S	86	50.89%	0	0.00%	0.001	0.497
R	83	49.11%	78	100.00%		

Drug resistance	Aminoglycosides		Glycopeptides		Fisher’s exact test p value	φ
	N	Percent	N	Percent		
S	86	50.89%	285	83.58%	0.001	0.346
R	83	49.11%	56	16.42%		

Drug resistance	Fluoroquinolones		Glycopeptides		Fisher’s exact test p value	φ
	N	Percent	N	Percent		
S	0	0.00%	285	83.58%	0.001	0.697
R	78	100.00%	56	16.42%		

Note: S, Susceptible, standard dosing regimen; I, Susceptible, increased exposure; R, Resistant; V, Cramér’s V.

### Klebsiella pneumonia

In the case of drug resistance of *Klebsiella pneumonia* to antibiotics group (see Table 4), the findings showed statistically significant differences between these groups of antibiotics in drug resistance frequency of *Klebsiella pneumonia* [χ^2^ (df = 6) = 303.44, p < 0.001, Cramér’s V = 0.221]. The effect size was moderate (see Table 4). In order to verify the differences between the analysed antibiotics group more precisely, comparisons between each pair of antibiotics groups were carried out. In this context, there was a statistically significant difference between Aminoglycosides and β-Lactams antibiotics group in the frequency of drug resistance to *Klebsiella pneumonia* [χ^2^ (df = 2) = 129.44, p < 0.001, Cramér’s V = 0.231]. The effect size was moderate. Considering the adjusted standardised residual values, differences between these antibiotic groups were observed for drug resistance categories such as susceptible -standard dosing regimen (S; z = 10.7; p < 0.001) and resistant (R; z = 11.3; p < 0.001). More precisely, more frequent drug resistance was for β-Lactams than for the Aminoglycosides antibiotics group (see Table 4). The findings showed the difference between the β-Lactams and Fluoroquinolones antibiotics group in the frequency of drug resistance to *Klebsiella pneumonia* [χ^2^ (df = 2) = 129.44, p < 0.001, Cramér’s V = 0.175]. The effect size was weak. Considering the adjusted standardised residual values (z > 4.5 and p < 0.001 for all), it can be indicated thatthe Fluoroquinolones antibiotics group was more often drug-resistant of *Klebsiella pneumonia* than β-Lactams antibiotics group (see Table 4). The pairwise comparisons analysis also showed the difference between β-Lactams and Fosfomycin G6PD in the frequency of drug resistance to*Klebsiella pneumonia* [χ^2^ (df = 2) = 63.75, p < 0.001, Cramér’s V = 0.173]. Considering the adjusted standardised residual values (z > 3.6 and p < 0.001 for all), it can be indicated that the β-Lactams antibiotics group was more often drug-resistant of *Klebsiella pneumonia* than the Fosfomycin G6PD antibiotics. However, it should be noted that the effect size was weak (see Table 4). There was a statistically significant difference between Fluoroquinolones and Aminoglycosides antibiotics group [χ^2^ (df = 2) = 240.11, p < 0.001, Cramér’s V = 0.497]. The effect size was relatively strong. Considering the adjusted standardised residual values (z > 4.9 and p < 0.001 for all), it can be pointed out that Fluoroquinolones were more often drug-resistant in relation to *Klebsiella pneumonia* than Aminoglycosides antibiotics group (see Table 4). The findings also showed a statistically significant difference between Aminoglycosides and Fosfomycin G6PD antibiotics group in the frequency of drug resistance to *Klebsiella pneumonia* [χ^2^ (df = 2) = 17.21, p < 0.001, Cramér’s V = 0.153]. The effect size was weak. Based on the adjusted standardised residual values, it can be pointed out that the difference was only in cases related to the susceptible - increased exposure (I; z = 3.9; p < 0.001). More precisely, the frequency of *Klebsiella pneumonia* susceptibility (increased exposure) was higher for Aminoglycosides than for the the Fosfomycin G6PD antibiotics (see Table 4). There was a statistically significant difference between Fluoroquinolones and Fosfomycin G6PD antibiotics [χ^2^ (df = 2) = 113.79, p < 0.001, Cramér’s V = 0.408]. The effect size was relatively strong. Considering the adjusted standardised residual values, differences between these antibiotic groups were observed for drug resistance categories such as susceptible - standard dosing regimen (S; z = 10.7; p < 0.001) and resistant (R; z = 10.5; p < 0.001). More precisely, more frequent drug resistance was for Fluoroquinolones than for the Fosfomycin G6PD antibiotics (see Table 4). All results are presented in Table 4.

**TABLE 4:** Difference between antibiotics group in drug resistance frequency for *Klebsiella pneumonia*.

Drug resistance	Aminoglycosides		β-Lactams		Fluoroquinolones		Fosfomycin w/G6PD		χ^2^	p	V
	N	Percent	N	Percent	N	Percent	N	Percent			
S	382	75.20%	932	48.69%	152	32.83%	168	76.36%	303.44	0.001	0.221
I	34	6.69%	107	5.59%	3	0.65%	0	0.00%			
R	92	18.11%	875	45.72%	308	66.52%	52	23.64%			

Pairwise comparisons															
Drug resistance	Aminoglycosides		β-Lactams		χ^2^	P	V	Drug resistance	β-Lactams		Fluoroquinolones		χ^2^	p	V
	N	Percent	N	Percent					N	Percent	N	Percent			
S	382	75.20%	932	48.69%	129.44	0.001	0.231	S	932	48.69%	152	32.83%	72.69	0.001	0.172
I	34	6.69%	107	5.59%				I	107	5.59%	3	0.65%			
R	92	18.11%	875	45.72%				R	875	45.72%	308	66.52%			

Drug resistance	β-Lactams		Fosfomycin w/G6PD		χ^2^	P	V	Drug resistance	Aminoglycosides		Fluoroquinolones		χ^2^	p	V
	N	Percent	N	Percent					N	Percent	N	Percent			
S	932	48.69%	168	76.36%	63.75	0.001	0.173	S	382	75.20%	152	32.83%	240.11	0.001	0.497
I	107	5.59%	0	0.00%				I	34	6.69%	3	0.65%			
R	875	45.72%	52	23.64%				R	92	18.11%	308	66.52%			

Drug resistance	Aminoglycosides		Fosfomycin w/G6PD		χ^2^	P	V	Drug resistance	Fluoroquinolones		Fosfomycin w/G6PD		χ^2^	p	V
	N	Percent	N	Percent					N	Percent	N	Percent			
S	382	75.20%	168	76.36%	17.21	0.001	0.153	S	152	32.83%	168	76.36%	113.79	0.001	0.408
I	34	6.69%	0	0.00%				I	3	0.65%	0	0.00%			
R	92	18.11%	52	23.64%				R	308	66.52%	52	23.64%			

Note: S, Susceptible, standard dosing regimen; I, Susceptible, increased exposure; R, Resistant; V, Cramér’s V.

### Proteus mirabilis

In the case of drug resistance of *Proteus mirabilis* to antibiotics group, the findings showed statistically significant differences between these groups of antibiotics in drug resistance frequency of *Proteus mirabilis* [Fisher-Freeman-Halton test = 93.84, p < 0.001, Cramér’s V = 0.207]. The effect size was moderate (see Table 5). In order to verify the differences between the analysed antibiotics group more precisely, comparisons between each pair of antibiotics groups were carried out. In this context, there was a statistically significant difference between Aminoglycosides and β-Lactams antibiotics group in the frequency of drug resistance to *Proteus mirabilis* [Fisher-Freeman-Halton test = 38.78, p < 0.001, Cramér’s V = 0.207]. The effect size was moderate. Considering the adjusted standardised residual values (z > 2.8 and p < 0.005 for all), it can be pointed out that Aminoglycosides were more often drug-resistant in relation to *Proteus mirabilis* than the β-Lactams antibiotics group (see Table 5). Similarly, there was a statistically significant difference between Fluoroquinolones and β-Lactams antibiotics group in the frequency of drug resistance to *Proteus mirabilis* [Fisher-Freeman-Halton test = 72.38, p < 0.001, Cramér’s V = 0.288]. In this context, Fluoroquinolones were more often drug-resistant to *Proteus mirabilis* than the β-Lactams antibiotics group. The effect size was moderate (see Table 5). All results are presented in Table 5.

**TABLE 5:** Difference between antibiotics group in drug resistance frequency for *Proteus mirabilis*.

Drug resistance	Aminoglycosides		β-Lactams		Fluoroquinolones		Fisher-Freeman-Halton exact test	p	V
	N	Percent	N	Percent	N	Percent			
S	114	59.07%	495	69.62%	83	50.30%	93.84	0.001	0.207
I	5	2.59%	80	11.25%	1	0.61%			
R	74	38.34%	136	19.13%	81	49.09%			

Pairwise comparisons							
Drug resistance	Aminoglycosides		β-Lactams		Fisher-Freeman-Halton exact test	p	V
	N	Percent	N	Percent			
S	114	59.07%	495	69.62%	38.78	0.001	0.207
I	5	2.59%	80	11.25%			
R	74	38.34%	136	19.13%			

Drug resistance	Fluoroquinolones		β-Lactams		Fisher-Freeman-Halton exact test	p	V
	N	Percent	N	Percent			
S	83	50.30%	495	69.62%	72.38	0.001	0.288
I	1	0.61%	80	11.25%			
R	81	49.09%	136	19.13%			

Drug resistance	Aminoglycosides		Fluoroquinolones		Fisher-Freeman-Halton exact test	p	V
	N	Percent	N	Percent			
S	114	59.07%	83	50.30%	5.51	0.062	0.126
I	5	2.59%	1	0.61%			
R	74	38.34%	81	49.09%			

Note: S, Susceptible, standard dosing regimen; I, Susceptible, increased exposure; R, Resistant; V, Cramér’s V.

### Pseudomonas aeruginosa

For drug resistance of *Pseudomonas aeruginosa*to antibiotics group, the results showed statistically significant differences between these groups of antibiotics in drug resistance frequency of *Pseudomonas aeruginosa* [Fisher-Freeman-Halton test = 330.35, p < 0.001, Cramér’s V = 0.408]. The effect size was relatively strong (see Table 6). In order to verify the differences between the analysed antibiotics group more precisely, comparisons between each pair of antibiotics groups were carried out. The pairwise comparisons analysis showed the difference between Aminoglycosides and β-Lactams antibiotics group in the frequency of drug resistance to *Pseudomonas aeruginosa* [Fisher-Freeman-Halton test = 278.02, p < 0.001, Cramér’s V = 0.558]. The effect size was relatively strong. Considering the adjusted standardised residual values, differences between Aminoglycosides and β-Lactamsantibiotics group were observed for drug resistance categories such as susceptible - standard dosing regimen (S; z = 13.6; p < 0.001) and susceptible -increased exposure (I; z = 13.5; p < 0.001). More precisely, the frequency of *Pseudomonas aeruginosa* susceptibility (increased exposure) was higher for β-Lactamsthan for the Aminoglycosides antibiotics group. Conversely, the frequency of *Pseudomonas aeruginosa* susceptibility (standard dosing regimen) was lower for β-Lactams than for the Aminoglycosides antibiotics group (see Table 6). There was also a statistically significant difference between the Fluoroquinolones and β-Lactams antibiotics group in the frequency of drug resistance to *Pseudomonas aeruginosa* [Fisher-Freeman-Halton test = 18.35, p < 0.001, Cramér’s V = 0.166]. Considering the adjusted standardised residual values, differences between these antibiotics groups were presented for drug resistance categories such as susceptible - standard dosing regimen (S; z = 3.8; p < 0.001) and resistant (R; z = 2.3; p = 0.021). More precisely, the Fluoroquinolones antibiotics group was more often drug-resistant of *Pseudomonas aeruginosa* than the β-Lactamsantibiotics group. However, it should be highlighted that the effect size was weak (see Table 6). There was a statistically significant difference between Aminoglycosides and Fluoroquinolones antibiotics group in the frequency of drug resistance to *Pseudomonas aeruginosa* [Fisher-Freeman-Halton test = 209.88, p < 0.001, Cramér’s V = 0.778]. The effect size was strong. Considering the adjusted standardised residual values (z > 2.6 and p < 0.009 for all), it can be indicated that the Fluoroquinolones antibiotics group was more often drug-resistant of *Pseudomonas aeruginosa* than the Aminoglycosides antibiotics group. Additionally, the frequency of *Pseudomonas aeruginosa* susceptibility (increased exposure) was higher for Fluoroquinolones than for the Aminoglycosides antibiotics group (see Table 6). Detailed results are shown in Table 6.

**TABLE 6:** Difference between antibiotics group in drug resistance frequency for *Pseudomonas aeruginosa*.

Drug resistance	Aminoglycosides		β-Lactams		Fluoroquinolones		Fisher-Freeman-Halton exact test	p	V
	N	Percent	N	Percent	N	Percent			
S	182	79.82%	125	25.67%	7	7.53%	330.35	0.001	0.408
I	1	0.44%	254	52.16%	55	59.14%			
R	45	19.74%	108	22.18%	31	33.33%			

Pairwise comparisons							
Drug resistance	Aminoglycosides		β-Lactams		Fisher-Freeman-Halton exact test	p	V
	N	Percent	N	Percent			
S	182	79.82%	125	25.67%	278.02	0.001	0.558
I	1	0.44%	254	52.16%			
R	45	19.74%	108	22.18%			

Drug resistance	Fluoroquinolones		β-Lactams		Fisher-Freeman-Halton exact test	p	V
	N	Percent	N	Percent			
S	7	7.53%	125	25.67%	18.35	0.001	0.166
I	55	59.14%	254	52.16%			
R	31	33.33%	108	22.18%			

Drug resistance	Aminoglycosides		Fluoroquinolones		Fisher-Freeman-Halton exact test	P	V
	N	Percent	N	Percent			
S	182	79.82%	7	7.53%	209.88	0.001	0.778
I	1	0.44%	55	59.14%			
R	45	19.74%	31	33.33%			

Note: S, Susceptible, standard dosing regimen; I, Susceptible, increased exposure; R, Resistant; V, Cramér’s V.

### Acinetobacter baumannii

For drug resistance of *Acinetobacter baumannii* to antibiotics group, the results showed statistically significant differences between these groups of antibiotics in drug resistance frequency of *Acinetobacter baumannii* [Fisher-Freeman-Halton test = 22.67, p < 0.001, Cramér’s V = 0.158]. It should be noted that the effect size was weak (see Table 7). In order to verify the differences between the analysed antibiotics group more precisely, comparisons between each pair of antibiotics groups were carried out. The pairwise comparisons analysis showed the difference between Aminoglycosides and β-Lactams antibiotics group in the frequency of drug resistance to *Acinetobacter baumannii* [Fisher-Freeman-Halton test = 7.25, p = 0.015, Cramér’s V = 0.151]. Considering the adjusted standardised residual values, differences between these antibiotics groups were presented for drug resistance categories such as susceptible - standard dosing regimen (S; z = 2.5; p = 0.012) and resistant (R; z = 2.6; p = 0.009). More precisely, the β-Lactamsantibiotics group was more often drug-resistant of *Acinetobacter baumannii* than the Aminoglycosides antibiotics group. However, it should be highlighted that the effect size was weak (see Table 7). There was a statistically significant difference between Fluoroquinolones and β-Lactams antibiotics group in the frequency of drug resistance to *Acinetobacter baumannii* [Fisher-Freeman-Halton test = 8.22, p = 0.007, Cramér’s V = 0.178]. Considering the adjusted standardised residual values, differences between these antibiotic groups were presented for drug resistance categories such as susceptible - standard dosing regimen (S; z = 2.3; p = 0.021). More precisely, *Acinetobacter baumannii* was more often susceptible (standard dosing regimen) to β-Lactams than the Fluoroquinolones antibiotics group. However, it should be highlighted that the effect size was weak (see Table 7). The pairwise comparisons analysis showed the difference between Aminoglycosides and Fluoroquinolones antibiotics group in the frequency of drug resistance to *Acinetobacter baumannii* [Fisher-Freeman-Halton test = 19.39, p < 0.001, Cramér’s V = 0.240]. The effect size was moderate. Considering the adjusted standardised residual values, differences between these antibiotics groups were presented for drug resistance categories such as susceptible - standard dosing regimen (S; z = 3.8; p < 0.001) and resistant (R; z = 3.2; p < 0.001). More precisely, the Fluoroquinolones antibiotics group was more often drug-resistant of *Escherichia coli* than the Aminoglycosides antibiotics group (see Table 7). The detailed results are shown in Table 7.

**TABLE 7:** Difference between antibiotics group in drug resistance frequency for *Acinetobacter baumannii*.

Drug resistance	Aminoglycosides		β-Lactams		Fluoroquinolones		Fisher-Freeman-Halton exact test	p	V
	N	Percent	N	Percent	N	Percent			
S	21	12.96%	8	5.00%	0	0.00%	22.67	0.001	0.158
I	1	0.62%	0	0.00%	2	1.92%			
R	140	86.42%	152	95.00%	102	98.08%			

Pairwise comparisons							
Drug resistance	Aminoglycosides		β-Lactams		Fisher-Freeman-Halton exact test	p	V
	N	Percent	N	Percent			
S	21	12.96%	8	5.00%	7.25	0.015	0.151
I	1	0.62%	0	0.00%			
R	140	86.42%	152	95.00%			

Drug resistance	Fluoroquinolones		β-Lactams		Fisher-Freeman-Halton exact test	p	V
	N	Percent	N	Percent			
S	0	0.00%	8	5.00%	8.22	0.007	0.178
I	2	1.92%	0	0.00%			
R	102	98.08%	152	95.00%			

Drug resistance	Aminoglycosides		Fluoroquinolones		Fisher-Freeman-Halton exact test	P	V
	N	Percent	N	Percent			
S	21	12.96%	0	0.00%	19.39	0.001	0.240
I	1	0.62%	2	1.92%			
R	140	86.42%	102	98.08%			

Note: S, Susceptible, standard dosing regimen; I, Susceptible, increased exposure; R, Resistant; V, Cramér’s V.

## Discussion

The proper selection of antibiotics for the treatment of infections is crucial for improving clinical outcomes. Any recommendations regarding the dosing of antimicrobial drugs should be applied with great caution, taking into account local epidemiology and patterns of pathogen resistance. Factors such as the severity of the disease, kidney function, coexisting conditions, and the patient’s response to treatment must be considered (Hoff et al., 2020).

Strains isolated from urinary tract infections in patients treated at the Mazovian Specialist Hospital showed varying sensitivity to the antibiotics used. One of the most frequently isolated pathogens was *Escherichia coli*, which exhibited a higher level of resistance to β-lactam antibiotics compared to aminoglycosides. This observation was also confirmed by other researchers. According to Akselsen et al., *E. coli* was 88% sensitive to ampicillin, 50% sensitive to gentamicin, and 42% sensitive to third-generation cephalosporins (Akselsen et al., 2022). In Poland, according to data from the National Antibiotic Protection Program (NPOA), the percentage of *E. coli* isolates resistant to aminopenicillins is high, while resistance to aminoglycosides remains at a lower level. According to NPOA data, in 2017, the resistance of *E. coli* strains isolated from urinary tract infections in Poland was 69.5% to aminopenicillins and 14% to aminoglycosides (Żabicka, 2018). Similar observations were made in other European countries. According to the 2022 data from the European Antimicrobial Resistance Surveillance Network (EARS-Net), an increase in the percentage of *E. coli* strains resistant to third-generation cephalosporins was observed in Germany and France, while resistance to aminoglycosides remained at a lower level. In Germany, the percentage of *E. coli* strains resistant to third-generation cephalosporins was around 10%, while resistance to aminoglycosides remained at about 5%. In France, the percentage of resistance to third-generation cephalosporins in *E. coli* strains was about 12%, while resistance to aminoglycosides was around 6% (European Centre for Disease Prevention and Control, 2023).

In Middle Eastern countries such as Saudi Arabia, a higher level of *E. coli* resistance to β-lactams compared to aminoglycosides has also been noted. Studies conducted in this region indicate a significant prevalence of *E. coli* strains producing extended-spectrum β-lactamases (ESBL), which leads to resistance to penicillins and cephalosporins. At the same time, resistance to aminoglycosides, although present, is less common. A study conducted in Saudi Arabia found that approximately 30% of *E. coli* strains isolated from urinary tract infections were resistant to third-generation cephalosporins, while resistance to aminoglycosides was about 15%. These data highlight the need for monitoring antibiotic resistance in this region and adjusting empirical therapy for urinary tract infections (Alhazmi et al., 2023).

Despite the limitations in the use of aminoglycosides, such as their potential nephrotoxicity, the lower level of resistance to this group of antibiotics makes them a valuable therapeutic option for urinary tract infections caused by β-lactam-resistant strains (Livermore, 2012).

An analysis of the available data confirms that *E. coli* strains causing urinary tract infections more often exhibit resistance to β-lactam antibiotics than to aminoglycosides, which is consistent with the results presented in this study.

Research conducted by Ruiz-Lievano and colleagues showed a systematic increase in resistance of *Escherichia coli* strains to fluoroquinolones in the United States, reaching an alarming level, especially in the context of hospital-acquired infections. This phenomenon is attributed to the extensive use of fluoroquinolones in both human and veterinary medicine, creating strong selective pressure that favors the development of resistant strains (Ruiz-Lievano et al., 2024).

Similar results were obtained in studies conducted in Europe. In the study by Azargun et al. (2020), it was noted that *E. coli* resistance to fluoroquinolones in urinary tract infections is increasing faster than resistance to β-lactams. The main mechanism underlying this resistance is the mutation of genes encoding DNA gyrase (gyrA) and topoisomerase IV (parC), which code for proteins involved in DNA replication and are the targets of fluoroquinolones (Azargun et al., 2020).

The phenomenon of increased resistance to fluoroquinolones may pose a therapeutic challenge, especially in regions where these antibiotics are often used as first-line drugs for urinary tract infections. Considering the data above, the use of fluoroquinolones should be carefully considered in justified cases, and alternative treatment strategies should be explored, especially in situations where strains are resistant to both classes of antibiotics.

Our own research, which indicates more frequent resistance of *E. coli* to fluoroquinolones than to β-lactams, aligns with reports from other authors, confirming the global trend of increasing resistance to fluoroquinolones in urinary tract infections.

The scientific literature increasingly supports the high sensitivity of *E. coli* to fosfomycin, even in strains resistant to β-lactams. A study by Mattioni Marchetti et al. (2023) showed that fosfomycin is effective against *E. coli* strains producing extended-spectrum β-lactamases (ESBL). Fosfomycin acts on bacteria at a different molecular level by blocking cell wall biosynthesis through inhibition of the UDP-N-acetylglucosamine enolpyruvyl transferase enzyme (MurA), which ensures its effectiveness against strains resistant to other classes of antibiotics (Mattioni Marchetti et al., 2023).

Studies conducted in Europe, including those by Falagas et al. (2016), also confirm the high sensitivity of *E. coli* to fosfomycin in the treatment of urinary tract infections. In regions where the percentage of *E. coli* strains resistant to β-lactams is increasing, fosfomycin is becoming an increasingly preferred therapeutic option, especially in outpatient treatment. Moreover, the use of fosfomycin as a single-dose drug for urinary tract infections is considered convenient and effective, which is an additional advantage in treating patients with uncomplicated urinary tract infections (Falagas et al., 2016).

It is worth emphasizing that in countries where fosfomycin is relatively rarely used, resistance to this antibiotic remains low, which makes it still effective against β-lactam-resistant *E. coli* strains. However, there are already early reports of the development of fosfomycin resistance mechanisms, primarily due to mutations in the murA gene or the presence of specific fosfomycinase enzymes, indicating the need to monitor the use of this antibiotic (Galindo-Méndez et al., 2022).

Additionally, studies by Saeed et al. (2021) also indicate that fosfomycin has a safe profile for use in patients with glucose-6-phosphate dehydrogenase (G6PD) deficiency because its metabolism does not lead to oxidation of red blood cells, which is crucial in this patient group (Saeed et al., 2021).

According to Falagas et al. (2016), fosfomycin can be used in a single dose, which reduces the risk of hemolysis in patients with G6PD deficiency, especially compared to long-term β-lactam therapies, which can pose a challenge in this patient population (Falagas et al., 2016). In the context of patients with G6PD deficiency who require alternative therapies, fosfomycin is a valuable option due to its safety profile and high antibacterial activity (Rodríguez-Gascón and Canut-Blasco, 2019).

Moreover, studies in Europe have shown that fosfomycin exhibits lower levels of resistance among *E. coli* strains than aminoglycosides. The study by Falagas et al. (2019) suggests that fosfomycin maintains high efficacy, making it an important drug, especially in the context of strains resistant to other classes of antibiotics (Falagas et al., 2019).

Further studies by Sojo-Dorado et al. (2022) confirm the increasing effectiveness of fosfomycin in the treatment of urinary tract infections. The authors emphasize that despite rare cases of fosfomycin resistance, its effectiveness in treating urinary tract infections remains high, which may be due to lower selective pressure compared to aminoglycosides, which are widely used in hospital-acquired infections. This highlights the need for further monitoring of resistance and consideration of fosfomycin as an alternative drug (Sojo-Dorado et al., 2022).

Our own research confirms that *E. coli* is more often resistant to β-lactams and aminoglycosides than to fosfomycin, which aligns with the literature. These findings have significant therapeutic implications, especially in patients with G6PD deficiency, for whom the use of fosfomycin may be a safe and effective alternative in treating urinary tract infections.

Studies indicate that resistance to aminoglycosides, although observed, develops more slowly in the *E. coli* population compared to fluoroquinolones. Aminoglycosides act on bacteria by binding to the 30S ribosomal subunit. In the study by Azargun et al. (2020) conducted in Europe, it was shown that *E. coli* strains causing urinary tract infections exhibit less resistance to aminoglycosides than to fluoroquinolones, which the authors attribute to the lower use of aminoglycosides in outpatient treatment (Azargun et al., 2020).

It should also be noted that the increasing resistance to fluoroquinolones in *E. coli* presents a serious therapeutic challenge, forcing clinicians to consider alternative options, such as aminoglycosides, especially in patients with uncomplicated urinary tract infections. However, due to potential side effects of aminoglycosides, such as nephrotoxicity and ototoxicity, their use must be carefully considered, and their long-term effects on the patient need to be monitored (Ruiz-Lievano et al., 2024).

The results of our own studies, which show that *E. coli* strains are more frequently resistant to fluoroquinolones than to aminoglycosides, are consistent with global trends. These findings confirm the need for continued monitoring of fluoroquinolone resistance and consideration of alternative therapies, particularly in the context of urinary tract infections caused by resistant strains.

Aminoglycosides, such as gentamicin and streptomycin, are used to treat enterococcal infections, often in combination with cell wall-targeting antibiotics to achieve a synergistic antibacterial effect. However, their effectiveness is limited due to the resistance mechanisms developed by *Enterococcus faecalis*. The most common resistance mechanism to aminoglycosides is the blocking of antibiotic binding to its target site, the 30S ribosomal subunit, as a result of the action of aminoglycoside-modifying enzymes (AME). Studies by Arias and Murray (2012) confirm that resistance to aminoglycosides among *E. faecalis* strains is becoming more widespread, limiting the effectiveness of this antibiotic class in treating urinary tract infections (Arias and Murray, 2012).

In the case of glycopeptides, such as vancomycin and teicoplanin, resistance mechanisms develop more slowly compared to aminoglycosides. Glycopeptides act by disrupting bacterial cell wall synthesis, making them effective against *E. faecalis*. The literature highlights that resistance to glycopeptides occurs less frequently and is mainly associated with *Enterococcus faecium* rather than *E. faecalis*. For this reason, glycopeptides remain a valuable option in the treatment of urinary tract infections caused by resistant *E. faecalis* strains (Guan et al., 2024).

Additionally, studies by Khalil et al. (2022) suggest that *E. faecalis* resistance to aminoglycosides is associated with frequent use of these antibiotics in the treatment of hospital-acquired infections, which creates selective pressure favoring the development of resistant strains. In contrast, the use of glycopeptides is often limited to severe infections, contributing to the slower development of resistance to this class of antibiotics (Khalil et al., 2022).

Fluoroquinolones, due to their broad spectrum of activity, have been used in the treatment of urinary tract infections. However, their effectiveness against enterococci is increasingly limited due to rising resistance. A study by Alhhazmi et al. (2024) showed that the use of fluoroquinolones is associated with the selection of resistant *E. faecalis* strains, and their use as first-line treatment for urinary tract infections contributes to the increase in resistance to these antibiotics (Alhhazmi et al., 2024).

In summary, the results of our own studies, indicating higher resistance of *E. faecalis* to aminoglycosides and fluoroquinolones compared to glycopeptides, are consistent with current scientific reports. These findings confirm the value of glycopeptides as a therapeutic option in infections caused by multidrug-resistant strains and emphasize the need to monitor resistance to ensure effective treatment of urinary tract infections caused by *E. faecalis*.

For *Enterococcus faecium*, the level of resistance to aminoglycosides is often lower than to fluoroquinolones. Studies by Arias and Murray (2012) indicate that resistance to aminoglycosides in *E. faecium* strains remains stable, partly due to the lower use of aminoglycosides in outpatient treatment (Arias and Murray, 2012).

Further research confirms that in the hospital environment, the use of fluoroquinolones promotes the selection of *E. faecium* strains resistant to these antibiotics, while aminoglycosides, due to their toxicological limitations, are used more sparingly. Mattioni Marchetti et al. (2023) note that the limited use of aminoglycosides may reduce selective pressure on the development of resistance, thus preserving their efficacy against certain *E. faecium* strains (Mattioni Marchetti et al., 2023).

The results of our own research, indicating higher resistance of *E. faecium* to fluoroquinolones than to aminoglycosides, are consistent with current literature reports. They confirm the need for careful use of fluoroquinolones in the treatment of infections caused by *E. faecium* and emphasize the importance of monitoring resistance, particularly in the context of multidrug-resistant strains.

In contrast to fluoroquinolones and aminoglycosides, resistance to glycopeptides in *E. faecium* develops much more slowly. The mechanism of glycopeptide resistance is mainly associated with the presence of the *vanA* and *vanB* genes, which alter the bacterial cell wall structure, preventing the binding of glycopeptides. In the studies by Arias and Murray (2012), resistance to glycopeptides in *E. faecium* was clearly lower compared to fluoroquinolones, indicating that the spread of vancomycin-resistant strains is more limited compared to resistance to fluoroquinolones (Arias and Murray, 2012). However, studies by Guan et al. (2024) suggest that while resistance to glycopeptides is significant, it is less common than resistance to aminoglycosides in *E. faecium* strains (Guan et al., 2024).

In summary, our results indicate that *E. faecium* strains are more often resistant to fluoroquinolones and aminoglycosides than to glycopeptides, which aligns with findings from other studies. The low levels of resistance to glycopeptides may be due to the more selective use of these antibiotics, especially in severe clinical cases and hospital-acquired infections. Belay et al. (2024) suggest that the controlled use of glycopeptides, limited mainly to cases of multidrug-resistant infections, helps slow the development of resistance among enterococci (Belay et al., 2024).

Resistance of *Klebsiella pneumoniae* to β-lactams is primarily associated with the production of extended-spectrum β-lactamases (ESBLs) and carbapenemases, which hydrolyze the β-lactam ring, rendering these antibiotics ineffective. Studies by Ramatla et al. (2023) confirm that ESBL- and carbapenemase-producing *K. pneumoniae* strains are increasingly prevalent in hospital settings, significantly limiting therapeutic options for treating hospital-acquired infections caused by these bacteria (Ramatla et al., 2023).

Further research suggests that the high rates of β-lactam resistance among *K. pneumoniae* strains result from selective pressure due to the frequent use of β-lactam antibiotics, both in hospitals and outpatient settings. Studies analyzing resistance in different regions of the world highlight that β-lactams remain one of the most commonly prescribed classes of antibiotics for treating bacterial infections, contributing to the rapid development of resistance (Mouanga-Ndzime et al., 2024).

Compared to β-lactams, aminoglycosides, such as amikacin and gentamicin, show higher effectiveness against *K. pneumoniae* strains. Although resistance to aminoglycosides does occur, it is less frequent and is often the result of enzymatic modification of the antibiotic or changes to the target site. Studies by Yang et al. (2023) demonstrated that in hospital-acquired *K. pneumoniae* strains, resistance to aminoglycosides is present but develops more slowly than resistance to β-lactams, which may be due to the more restricted use of aminoglycosides (Yang et al., 2023). Additionally, studies by Maraki et al. (2024) indicate that aminoglycosides maintain higher effectiveness against *K. pneumoniae* compared to fluoroquinolones (Maraki et al., 2024).

The results of our own studies, which show more frequent resistance of *K. pneumoniae* to β-lactams and fluoroquinolones than to aminoglycosides, align with the latest scientific reports. These findings confirm that the limited use of aminoglycosides may help preserve their effectiveness, making them an important therapeutic alternative, particularly in the context of infections caused by β-lactam-resistant strains. These results emphasize the need for continued research into resistance and the monitoring of antibiotic usage to optimize treatment strategies and reduce the growing resistance.

Studies by Rezaei et al. (2024) and Geetha et al. (2020) showed that resistance to fluoroquinolones is becoming more common in *K. pneumoniae* strains, especially in hospital settings (Rezaei et al., 2024; Geetha et al., 2020). This increase may be linked to the widespread use of fluoroquinolones as first-line treatments for urinary tract infections and other bacterial infections. Further studies show that fluoroquinolone resistance is more common and develops faster compared to β-lactams, which may result from selective pressure caused by frequent use of fluoroquinolones. Studies by Maraki et al. (2024) suggest that the widespread use of fluoroquinolones, especially in outpatient settings, promotes the selection of fluoroquinolone-resistant strains, while resistance to β-lactams, although significant, develops more slowly (Maraki et al., 2024).

An analysis by Ramatla et al. (2023) suggests that reducing the frequency of fluoroquinolone use in cases where they are not essential could help limit the growing resistance to this drug class (Ramatla et al., 2023).

Fosfomycin remains effective against many *K. pneumoniae* strains, even those resistant to β-lactams or fluoroquinolones. Its unique mechanism of action makes it a valuable alternative for treating urinary tract infections, particularly in cases of multidrug resistance. In contrast to fluoroquinolones, fosfomycin is less commonly used, resulting in less selective pressure, which helps preserve the drug’s effectiveness. Studies by Leelawattanachai et al. (2020) indicate that fosfomycin remains effective against *K. pneumoniae* strains producing extended-spectrum β-lactamases (Leelawattanachai et al., 2020).

In summary, our results indicate that *K. pneumoniae* is more frequently resistant to fluoroquinolones than to β-lactams or fosfomycin, which aligns with the latest scientific reports. These findings emphasize the need for the restricted use of fluoroquinolones and highlight the value of fosfomycin as an effective therapeutic alternative in cases of infections caused by multidrug-resistant strains.

Among *Proteus mirabilis* strains, resistance to aminoglycosides is primarily due to the presence of aminoglycoside-modifying enzymes (AMEs). These mechanisms lead to the rapid development of resistance to drugs such as gentamicin and amikacin. Studies by Vaez et al. (2022) show that AME enzymes are frequently found in *P. mirabilis* strains, contributing to the high level of resistance to aminoglycosides in this population (Vaez et al., 2022).

In contrast, β-lactams, which act by inhibiting bacterial cell wall synthesis, remain more effective against *P. mirabilis*. In this species, β-lactamases are less common or exhibit lower activity compared to the mechanisms responsible for aminoglycoside resistance. Research by ElTaweel et al. (2024) showed that *P. mirabilis* is more sensitive to β-lactams, especially cephalosporins, suggesting that antibiotics in this class are a more effective treatment option for infections caused by this pathogen (ElTaweel et al., 2024).

It is important to note that the use of β-lactams as first-line antibiotics for treating *P. mirabilis* infections is supported by their relatively low resistance levels. In contrast to aminoglycosides, which are prone to enzymatic resistance, β-lactams remain effective in a wide range of infections caused by this species. At the same time, studies highlight the need for cautious use of aminoglycosides in treating *P. mirabilis* infections and recommend monitoring their effectiveness.

Compared to fluoroquinolones, β-lactams have diverse mechanisms of action and are not subject to mutations in the same target genes. Resistance to β-lactams in *P. mirabilis* is primarily associated with the production of β-lactamases, but these enzymes are less prevalent in hospital strains than mutations leading to resistance to fluoroquinolones. Research by Rubic et al. (2021) confirms that *P. mirabilis* exhibits lower levels of resistance to β-lactam antibiotics, making them an effective option for treating infections caused by this pathogen (Rubic et al., 2021).

Our own study results show that *P. mirabilis* is more frequently resistant to aminoglycosides and fluoroquinolones than to β-lactams, which is consistent with the scientific literature. These findings underscore the importance of using β-lactams as the preferred therapeutic option for treating *P. mirabilis* infections and the need to monitor resistance to aminoglycosides and fluoroquinolones to prevent the development of multidrug-resistant strains.

*Pseudomonas aeruginosa* strains, due to the extensive use of fluoroquinolones, quickly develop resistance to this class of drugs. In addition to mutations in the *gyrA* and *parC* genes, *P. aeruginosa* may also develop resistance to fluoroquinolones through the activation of efflux pumps, such as MexAB-OprM, which actively expel the drug from the cell, and by reducing the permeability of the outer membrane, limiting the entry of the antibiotic into the cell. Studies by Thompson et al. (2024) and Shariati et al. (2022) demonstrated that these mechanisms are common in *P. aeruginosa* strains resistant to fluoroquinolones, making this drug class less effective in urinary tract infections (Thompson et al., 2024; Shariati et al., 2022).

The high resistance to fluoroquinolones in *P. aeruginosa* is of clinical importance because it limits the effectiveness of this drug class in treating infections, especially hospital-acquired ones, which are often caused by multidrug-resistant strains. Therefore, cautious use of fluoroquinolones is recommended, and alternative options, such as β-lactams or their combinations with inhibitors, should be considered to slow the development of resistance.

In the case of β-lactams, such as piperacillin/tazobactam, ceftazidime, or meropenem, resistance in *P. aeruginosa* develops more slowly and to a lesser extent. The mechanisms of resistance to β-lactams include the production of β-lactamases, such as carbapenemases (e.g., OXA and VIM enzymes), which hydrolyze the β-lactam ring and inactivate the antibiotic. Nevertheless, β-lactams often remain effective against *P. aeruginosa*, especially when used in combinations that may include β-lactamase inhibitors. Studies by Schwartz et al. (2024) showed that β-lactams retain higher effectiveness against *P. aeruginosa* compared to fluoroquinolones, making them a valuable therapeutic option for treating infections caused by this pathogen (Schwartz et al., 2024).

Our own research findings, which show that *P. aeruginosa* is more frequently resistant to fluoroquinolones than to β-lactams, are consistent with other studies. These findings highlight the need for the rational use of fluoroquinolones and continued monitoring of resistance to ensure the effectiveness of therapy against infections caused by *P. aeruginosa*.

*Pseudomonas aeruginosa* develops resistance to aminoglycosides through aminoglycoside-modifying enzymes (AMEs); however, the level of this resistance is generally lower compared to fluoroquinolones. Studies by López Montesinos et al. (2022) showed that aminoglycosides, especially amikacin, retain higher effectiveness against *P. aeruginosa* strains in urinary tract infections. This may be due to the more limited use of these antibiotics and less selective pressure (López Montesinos et al., 2022).

Resistance of *Acinetobacter baumannii* to β-lactams, including penicillins and carbapenems, is the result of complex mechanisms such as the production of β-lactamases (e.g., OXA carbapenemases), reduced membrane permeability, and activation of efflux pumps. In particular, the production of OXA-type carbapenemases, such as OXA-23 and OXA-58, is commonly found in *A. baumannii* strains and contributes to high levels of carbapenem resistance, making carbapenems, among the most effective β-lactams for treating multidrug-resistant infections, less useful. Research by Castanheira et al. (2023) showed that *A. baumannii* strains causing urinary tract infections often exhibit resistance to β-lactams, significantly limiting therapeutic options (Castanheira et al., 2023).

Although *A. baumannii* can also show resistance to aminoglycosides, especially through enzymatic modification of the antibiotics, the level of this resistance is often lower than that seen with β-lactams or fluoroquinolones. Studies conducted by various research groups suggest that aminoglycosides, particularly amikacin, maintain relatively high effectiveness against *A. baumannii* strains isolated from urinary tract infections. This may be due to the more limited use of aminoglycosides and less selective pressure compared to fluoroquinolones (Zohar et al., 2024; Gharaibeh et al., 2024; Rafailidis et al., 2024).

In the context of treating *A. baumannii* infections, especially urinary tract infections, the choice of antibiotics should be based on the results of susceptibility testing. High resistance to β-lactams indicates the need for cautious use of this antibiotic class and consideration of aminoglycosides or combination therapy to enhance treatment effectiveness and prevent further development of resistance.

Resistance of *A. baumannii* to fluoroquinolones, such as ciprofloxacin and levofloxacin, is the result of several mechanisms. Primarily, this involves mutations in the *gyrA* and *parC* genes. Additionally, *A. baumannii* possesses efflux pumps such as AdeABC, which effectively expel fluoroquinolones from the bacterial cell, contributing to their reduced efficacy. Studies by Aboelenin et al. (2024) and Kherroubi et al. (2024) show that these resistance mechanisms are widely spread among *A. baumannii* strains, limiting the effectiveness of fluoroquinolones, especially in hospital settings (Aboelenin et al., 2024; Kherroubi et al., 2024).

Research by Scoffone et al. (2025) confirms that in some cases, β-lactams are more effective than fluoroquinolones for treating *A. baumannii*-caused infections, making them an important therapeutic tool (Scoffone et al., 2025).

In conclusion, the selection of antibiotic therapy in urinary tract infections depends on the specific resistance profile of the pathogen involved. For infections caused by *Escherichia coli*, *Klebsiella pneumoniae*, *Proteus mirabilis*, *Pseudomonas aeruginosa*, and *Acinetobacter baumannii*, resistance to various antibiotic groups, such as β-lactams, aminoglycosides, and fluoroquinolones, poses a significant clinical challenge (Abayneh et al., 2023; Nelson et al., 2024; Murray et al., 2022).

For *Escherichia coli* and *Klebsiella pneumoniae* strains, β-lactam antibiotics, particularly cephalosporins and carbapenems, often remain effective, although widespread use of these drugs contributes to increasing resistance, primarily due to β-lactamase production. In strains resistant to β-lactams, fosfomycin demonstrates high efficacy, especially in outpatient treatment and in patients with G6PD deficiency who are at risk of hemolytic reactions from other antibiotics (Romyasamit et al., 2024).

In the treatment of infections caused by *Pseudomonas aeruginosa* and *Acinetobacter baumannii*, often multidrug-resistant pathogens, the choice of antibiotics is more limited. *P. aeruginosa* shows higher resistance to fluoroquinolones compared to β-lactams, making carbapenems and β-lactam combinations with β-lactamase inhibitors more effective therapeutic options (Morris et al., 2019; Zheng et al., 2022). Similarly, *A. baumannii* demonstrates higher resistance to fluoroquinolones and β-lactams than to aminoglycosides, making the latter a useful alternative, although their use is limited due to potential toxicity (Jeong et al., 2024).

Aminoglycosides such as amikacin and gentamicin remain effective options in the treatment of infections caused by strains resistant to fluoroquinolones and β-lactams, especially *Acinetobacter baumannii*. However, their use requires caution and monitoring due to the potential nephrotoxic and ototoxic effects (Adeniji et al., 2022; Alqurashi et al., 2022; Rafailidis et al., 2024; Thy et al., 2023).

These data highlight the importance of individualizing therapy based on the results of susceptibility testing, particularly in hospital settings, where there is significant selective pressure that favors the development of resistant strains. Proper antibiotic use, resistance monitoring, and a rational approach to antibiotic therapy can help limit the development of resistance and improve the effectiveness of treatment for urinary tract infections caused by multidrug-resistant pathogens.

## Conclusion

In conclusion, the problem of antibiotic resistance in urinary tract infections treated in hospital settings requires a multifaceted approach, including regular resistance monitoring, responsible antibiotic use, therapy individualization, and the search for and use of alternative drugs. These actions are crucial for ensuring the effectiveness of treatment and preventing the further development of resistant pathogens.

## Data Availability

The original contributions presented in the study are included in the article/supplementary material, further inquiries can be directed to the corresponding authors.
